# Mechanical ventilation in emergency departments: Non invasive or invasive mechanical ventilation. Where is the answer?

**DOI:** 10.1186/1757-7241-20-40

**Published:** 2012-06-26

**Authors:** Antonio M Esquinas Rodriguez, Roberto Cosentini, Peter J Papadakos

**Affiliations:** 1 International Fellow AARC, Intensive Care Unit, Hospital Morales Meseguer, Avenida Marques de Los Velez s/n, Murcia, 30500, Spain; 2 Gruppo_NIV UO Medicina d'Urgenza, Fondazione Ca' Granda, IRRCS Policlinico Milano, Via Francesco Sforza, 35, Milan, Italy; 3 Surgery and Neurosurgery, Director Critical Care, University of Rochester, Rochester, NY, USA

**Keywords:** Emergency department, Non-invasive mechanical ventilation, Mechanical ventilation, Trauma, Length of stay

## Abstract

The Emergency Department length of stay for patients requiring mechanical ventilation paper in this issue is very illustrative of many variables that still confound the way we treat patients that may not require endotracheal intubation (ETI) but may benefit from non-invasive mechanical ventilation (NIV) [1].

## Background

Rose L et al. bring to light several important aspects the chief of which is the way we view this modality [1]. Patients that refused endotracheal intubation, but accepted NIV were not clearly identified as to whether this was done as a terminal mode as an end-of-life decision or if this was a bridge to prevent endotracheal intubation and allow pharmacotherapy to improve patient status such as heart failure. Caregiver trends and service battles are clearly illustrated in this study as a large confounder was or which service will care for the patient on the floor, step down or intensive care unit (ICU). With the great pressure to provide proper utilization of staff and technology rich environments, much of the push back to keep patients of NIV in the in the ED may be from the ICU itself. ED staff input is also a key variable leading the delays we found in interesting that only 55.2% of patients received arterial blood gas following commencement of mechanical ventilation which would be unusual for accepted ICU practice [1]. This lack of follow-up illustrates how patients receiving ventilator support are placed in a holding pattern in the ED and do not get active care input.

The core pattern illustrated by this study is the reluctance of EDs and ICUs to develop set protocols to care for these patients and provide rapid placement. The trauma data in this study supports this statement in that trauma centers utilize the standards of the advance trauma life support guidelines most meet quality indicators for time to transfer either to the ICU or the operative theater. The other important point is that ED staff may not fully appreciate the status of a patient on NIV [1]. The intubated patient is always considered a patient that requires critical care, thus staff may pressure the ICU to spread transfer [1,2]. It would be interesting for these investigators recorded the number of calls made to units by staff.

We believe that many of the delays could be addressed through developments of guidelines or protocols on how we care for both intubated and NIV patients in the emergency department [3,4]. Also, resources such as RT’s or specially trained nurses need to be placed in the ED to manage these patients so they do not fall behind in their care. This is an important aspect in providing ideal care, especially in patients where NIV is being used to prevent intubation [3,4]. Delays in transfer to units may increase as economic and acuity pressures add to the burdens of the health care system [5,6]. So we much address this issue o ventilated patients before it balloons and affects mortality and care indicators.

## Abbreviation

ED, Emergency Department; ETI, Endotracheal intubation; ICU, Intensive Care Unit; NIV, Non-invasive mechanical ventilation.

## Competing of interest

The authors should be congratulated for bringing this data to light. The author(s) declare have no potentially conflicting personal of financial interests.
